# The rate of publication of free papers at the 2008 and 2010 European Society of Sports Traumatology Knee Surgery and Arthroscopy congresses

**DOI:** 10.1186/s40634-017-0090-8

**Published:** 2017-05-10

**Authors:** Jeffrey Kay, Muzammil Memon, Joelle Rogozinsky, Darren de SA, Nicole Simunovic, Romain Seil, Jon Karlsson, Olufemi Rolland Ayeni

**Affiliations:** 1 Michael G. DeGroote School of Medicine, McMaster University, Hamilton, ON Canada; 2 Department of Medicine and School of Physical and Occupational Therapy, McGill University, Montreal, Canada; 3 Division of Orthopaedic Surgery, Department of Surgery, McMaster University, Hamilton, Canada; 4 Department of Clinical Epidemiology and Biostatistics, McMaster University, Hamilton, ON Canada; 5 Département de l’Appareil Locomoteur, Centre Hospitalier de Luxembourg, Luxembourg, Luxembourg; 6 Department of Orthopaedics, Sahlgrenska University Hospital, Mölndal, Sweden; 7 Division of Orthopaedic Surgery, Department of Surgery, McMaster University Medical Centre, 1200 Main St West, 4E15, Hamilton, ON L8N 3Z5 Canada; 8 Sports Medicine Research Laboratory, Luxembourg Institute of Health, Luxembourg, Luxembourg

**Keywords:** Evidence-based medicine, ESSKA congress, Publication rate, Level of evidence

## Abstract

**Background:**

The purpose of this study was to evaluate the frequency with which free papers presented at the 2008 and 2010 European Society of Sports Traumatology Knee Surgery and Arthroscopy (ESSKA) congress were ultimately published in peer-reviewed journals. Moreover, this study evaluated whether any correlations exist between the level of evidence of the free papers and their frequency of publication or the impact factor of the journals in which they are published.

**Methods:**

Free papers presented at the 2008 and 2010 ESSKA congresses were included for assessment. Clinical papers (observational studies and trials involving direct interaction between an investigator and human subjects) were graded for level of evidence by two independent reviewers. A comprehensive strategy was used to search the databases PubMed, Ovid (MEDLINE), and EMBASE for all publications corresponding to the included free papers.

**Results:**

Three hundred-ninety presentations were evaluated, of which 215 (55%) were ultimately published in a peer-reviewed journal within five years of the presentation date. The mean time from presentation to publication was 16 months (SD 25 months). There was no significant difference in the distribution of the level of evidence between studies that were ultimately published, versus those that were not published (n.s.). The level of evidence of the published study was not a significant predictor of the impact factor of the journal in which it was published (n.s.). Presentations were most commonly published in *Knee Surgery*, *Sports Traumatology*, *Arthroscopy* (24%) and *The American Journal of Sports Medicine* (22%).

**Conclusion:**

Free papers at the 2008 and 2010 ESSKA congress were published at a frequency that is comparable to that at other orthopaedic meetings. The publication rate was similar across all levels of evidence. Further encouragement of manuscript preparation and submission following these meetings could help to ensure important research findings are disseminated to large audiences.

## Background

To ensure that important research findings ultimately have an impact on clinical decisions, their timely dissemination to target audiences is critical. Often the results of the latest research trials are initially presented at scientific meetings before being published as full-text articles in peer-reviewed journals. The European Society of Sports Traumatology, Knee Surgery and Arthroscopy (ESSKA) biannual congress is one of the major venues for leading orthopaedic surgeons specializing in degenerative joint disease and sports medicine to present and learn about the latest techniques and research in these fields. The level of evidence of research presented at the ESSKA congress has demonstrated significant improvement over the past decade, with a high proportion of level I studies being presented (Kay et al. 2016a). While these meetings provide its attendees with important information in terms of the latest advancements, the findings that are presented are not usually implemented into clinical practice until after they are published in peer-reviewed journals and disseminated to much larger audiences (de SA et al. 2015). It is therefore critical that these presentations are followed up with manuscript preparation, and ultimately publication. The publication rate of presentations at other orthopaedic meetings such as the American Academy of Orthopaedic Surgeons (AAOS) (49%), Arthroscopy Association of North America (AANA) (55%) and the American Shoulder and Elbow Surgeons (ASES) (49%), suggest that a significant proportion of presentations at these meetings may not ultimately be widely disseminated to impact clinical decisions (Kay et al. 2016b; Kay et al. 2016c; Voleti et al. 2013). Moreover, there are conflicting results with respect the impact of the level of evidence of the presentations on the publication rate at these meetings. The publication rate of presentations at the ESSKA congress has not been evaluated to date.

The purpose of this study was to evaluate the frequency with which free papers presented at the 2008 and 2010 European Society of Sports Traumatology Knee Surgery and Arthroscopy (ESSKA) Congress were ultimately published in a peer-reviewed journal. Moreover, this study evaluated whether any correlations exist between the level of evidence of the free papers and their frequency of publication or the impact factor of the journals in which they are published.

## Methods

### Study eligibility and assessment

The methodology used in the present study is similar to that used in a previous study to assess the publication rate of presentations at the American Association of North America (AANA) meetings (Kay et al. 2016c). Inclusion criteria included free papers presented at the 2008 and 2010 ESSKA congresses, as these years would allow for evaluation of all corresponding papers published within five years of presentation. The abstracts of the free papers presented at the ESSKA biannual congresses have been published electronically by Knee Surgery, Sports Traumatology, Arthroscopy (KSSTA). Two reviewers (J.R. & M.M.) comprehensively searched PubMed, MEDLINE, and EMBASE using a modified version of the search strategy described by Bhandari et al. (Bhandari et al. 2002). For each free paper presented, the first, second, and last author were searched using these databases. Next, the Boolean operator ‘AND’ was used to combine this search with additional key words from the title or abstract of the study until the search yielded only one result. If the identified publication was published less than five years after the date of presentation, this publication was recorded and its information was abstracted. A 5-year time period was chosen as previous studies have demonstrated satisfactory accuracy using this time frame (Bhandari et al. 2002). All peer-reviewed journals, including open access journals, were included in the assessment. The abstracts were then screened independently and in duplicate by the reviewers for those deemed as clinical studies. More specifically the free papers were assessed for any trial or observational study that included direct interaction between an investigator and their human subjects. While these studies may ultimately have important clinical implications, they are unable to be classified into a level of evidence according to the AAOS classification scheme (Wright 2005). The included abstracts were then graded by the two reviewers independently for level of evidence using the AAOS classification scheme (Wright 2005).

### Data extraction and statistical analysis

The relevant study data was extracted from the included free papers including the title, authors, location, sample size, journal of publication (if applicable), and joint of focus. These data were recorded in Microsoft Excel 2015 spreadsheets (Microsoft, Redmond, WA, USA). The Web of Science database was used to obtain the impact factors of each journal. In order to account for variation by year, the impact factor corresponding to the year of publication were used for the analysis. Kappa (κ) statistics were determined for the abstract screening and level of evidence evaluation stage to assess inter-reviewer agreement. The proportions of free papers that were published was determined by level of evidence and year of presentation. Means and standard deviations were calculated for the time to publication and impact factors. Chi-squared tests were used in order to test for changes in the proportion of published free papers and student t-tests as well as one way ANOVA tests were used when comparing the mean values of quantitative data. A *p*-value of 0.05 or less was considered to be significant. All statistics were calculated using Minitab ® statistical software version 17 (Minitab Inc., State College, USA).

## Results

**Fig. 1:**
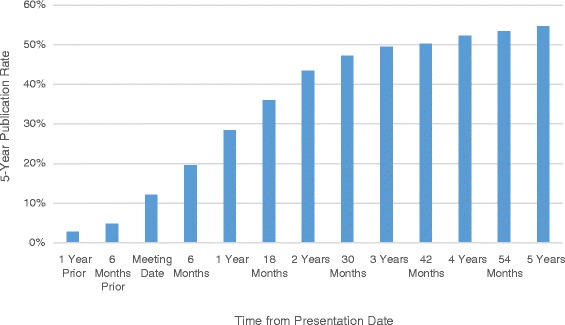
Cumulative graph demonstrating the frequency with which presentations at the ESSKA congress were ultimately published at various points in time from the meeting dates

Overall, all 390 free papers presented in 2008 and 2010 were assessed. There was almost perfect agreement at the screening, and level of evidence evaluation stages with κ (and 95% confidence intervals) of 0.96 (0.90, 1.00) and 0.87 (0.82, 0.93), respectively. In total, 215 (55%) of the free papers were ultimately published in peer reviewed journals within 5 years of the presentation date. Forty-six (12%) studies were published before the date they were presented. The mean time from presentation to publication was 16 months (SD = 25 months) (Fig. 1).

**Fig. 2:**
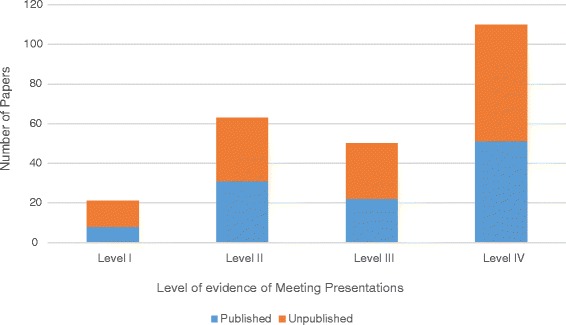
The number of papers presented and ultimately published by level of evidence

**Fig. 3:**
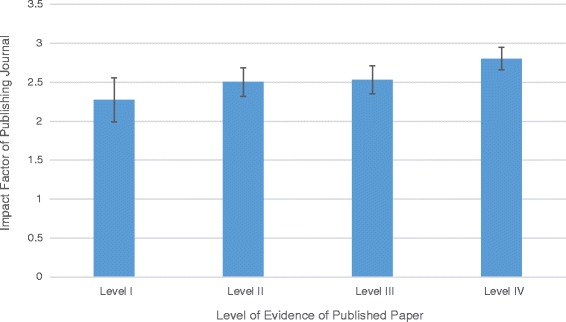
Mean ± SEM impact factor of the publishing journals by level of evidence of the published study

The 5-year publication rate in 2010 (113/192, 59%) was slightly higher than the publication rate in 2008 (102/198, 52%) (*p* = n.s.). Two hundred-fifty studies were considered clinical and assigned a level of evidence. Of these studies, 117 (47%) were published in a peer reviewed journal within 5 years of presentation. There was no significant difference in the distribution of the level of evidence between studies that were ultimately published, versus those that were not published (*p* = n.s.) (Fig. 2). The level of evidence of the published study was not a significant predictor of the impact factor of the journal in which it was published (*p* = n.s.) (Fig. 3).

**Fig. 4:**
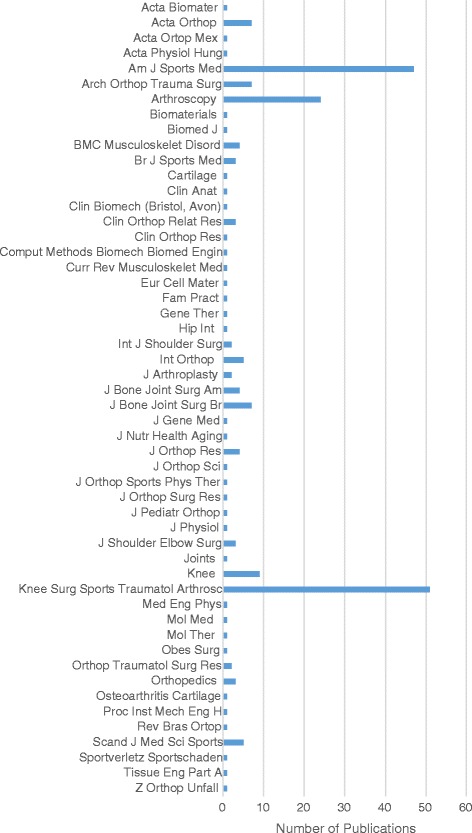
Productivity of each journal with respect to the total number of papers published

The papers were ultimately published in 52 different journals. Fifty-one (24%) studies were published in *Knee Surgery*, *Sports Traumatology, Arthroscopy*, 47 (22%) were published in *The American Journal of Sports Medicine,* 24 (11%) were published in *Arthroscopy*, and 9 (4%) were published in *The Knee* (Fig. 4).

## Discussion

The most important finding in the present study was identifying that more than half of the presentations at the 2008 and 2010 ESSKA congresses have ultimately been published in peer-reviewed journals. There was no effect of level of evidence on the publication rate, with similar frequencies of presentations published across all levels of evidence. Moreover, there was no association between the level of evidence and the impact factor of the journal in which the study was published. Presentations were most commonly published in *Knee Surgery, Sports Traumatology, Arthroscopy* followed by *The American Journal of Sports Medicine.*

The critical concepts of knowledge translation involve the synthesis and exchange of ethically and methodologically sound research in order to improve the health of patients (Graham et al. 2006). To accomplish these important goals, it is vital that important research findings are disseminated to large audiences so that they have the opportunity to influence clinical decisions. Publication of research findings in recognized peer-reviewed journals is a requisite step in this process. While the publication rate of presentations at the ESSKA congress has not yet been evaluated to date, the rate of publication of presentations at other orthopaedic meetings have been previously studied. The publication rate of presentations at the AAOS meetings was assessed for the time periods 1990–1992, 1993, 1996, and 2001. The publication rates of presentations over those time periods were respectively reported as 46%, 44%, 34%, and 49% (Voleti et al. 2013; Bhandari et al. 2002; Hamlet et al. 1997; Murrey et al. 1999). The publication rate of presentations at the Society of Military Orthopaedic Surgeons meetings from 1999 to 2003 was reported as 44% (Fuller et al. 2012). Presentations at the 1997 and 1999 International Society of Arthroscopy, Knee Surgery & Orthopaedic Sports Medicine (ISAKOS) meetings were published with frequencies of 35% and 39% respectively (Eck 2005). The publication rate of 55% identified in the present study is greater than, although comparable, to these previously reported rates. Overall, 12% of the presentations at the ESSKA congresses had been published before the date of the presentation. This number may be one indication of the originality of the presentations at a meeting. This number is slightly higher than the 3% and 7% of papers that were published before their corresponding presentations at the 2005–2010 ASES and 2006–2010 AANA meetings, respectively. One possible explanation for this discrepancy may relate to the bi-annual nature of the ESSKA congress which allows a longer time period for the presentations to get published before they are actually presented (Kay et al. 2016b; Kay et al. 2016c).

Conflicting results have been reported with respect to the association between the level of evidence and the publication rate of presentations at orthopaedic meetings. Voleti at al. reported a significantly improved publication rates in presentations of higher levels of evidence at the 2001 AAOS meeting (Voleti et al. 2013). On the other hand, no such correlation was identified at either the 2006–2010 AANA meetings or at the 2005–2010 ASES meetings (Kay et al. 2016b; Kay et al. 2016c). The present study found no association between the level of evidence and the publication rates of free papers presented at the 2008 and 2010 ESSKA meetings. The level of evidence of a study is one method of screening its quality. The assessment of level of evidence in orthopaedic research has been published by the AAOS, in a system adopted by that described in *the Journal of Bone and Joint Surgery* (Wright 2005). The system assigns a level of evidence to each study with methodological designs such as high quality randomized controlled trials graded as a level I, whereas case series’ and reports are assigned a level IV. The idea of such a system would be that research that has been conducted using study designs that inherently limit the chance of bias would be more likely to have meaningful clinical impact than research conducted using a study design that is less rigorous. However, Marx et al. have demonstrated that the level of evidence of a paper may not provide a direct reflection of its clinical importance (Marx et al. 2015).

This study is the first to evaluate the frequency with which presentations at the ESSKA biannual congress have been published in peer-reviewed journals. Strengths of this study include the use of multiple databases and an almost perfect level of inter-reviewer agreement at both the screening (κ = 0.96) and level of evidence evaluation (κ =0.87) stages. Although multiple databases were used, there is a possibility that some full-text publications were not identified by the search strategy, particularly if the title of the presentation had changed when the manuscript was being written. Thus, the publication rate identified by the present study is likely lower than the true frequency with which presentations at the ESSKA congress are published. However, a change in title would likely have a small effect on the ability to identify the project, considering the search strategy involved searching the first and last authors of the study rather than the title. Moreover, the results are encouraging considering a similar publication rate to that identified at other orthopaedic meetings (Kay et al. 2016b; Kay et al. 2016c). The methodological quality of the study design used by the presentations was assessed based on the abstracts alone. Given the word constraints inherent in the short abstracts, the assessment in the present study may not have provided a completely thorough representation of the methodological quality of the studies. Although it has been found that some orthopedic presentations are included at multiple meetings, (Bhandari et al. 2005) the present study only considered the presentations at the ESSKA congress. It is possible that presentations at multiple meetings could impact the quality of the research being presented. Non-clinical studies including cadaveric studies, animal studies and technique demonstrations were not included for assessment in the present study. Although these studies provide critical information for those attending the ESSKA congresses, and they may ultimately have important clinical impact, they cannot be graded for level of evidence using the AAOS classification system and would not provide for a meaningful comparison.

Although the proportion of free papers presented at the 2008 and 2010 ESSKA congress that were ultimately published is similar to the frequency identified at other orthopaedic meetings, about half of the free papers that were presented remain unpublished. Moreover, there was no association between the level of evidence of the free papers and the rate at which the presentations were published. One possible explanation for these findings would be that the free papers that are unpublished are not, in fact, being rejected for publications, but rather they are not being submitted as full-text manuscripts to peer-reviewed journals by the respective authors. Sprague et al. studied presentations that remained unpublished after AAOS meetings, and found that only 25% of these studies were rejected for publication by a peer-reviewed journal (Sprague et al. 2003). The other 75% of unpublished papers were never submitted for publication, with the most commonly given reasons being “insufficient time to prepare a manuscript”, “co-authors moving or changing institutions”, and “manuscript still in progress” (Sprague et al. 2003). If similar factors have been preventing the authors of free papers from the ESSKA congresses to prepare manuscripts for submission, there may be important clinical findings that are never disseminated to large audiences and impacting clinical decisions. It is therefore important to ensure that the latest research findings presented at the ESSKA congress are submitted for peer-reviewed publication to allow for the widespread dissemination of the highest quality research. One potential policy that might, if implemented, improve the publication rate of presentations at the ESSKA congress involves encouraging the submission of a complete manuscript accompanying each abstract or within a set period of time following each presentation. Future research might evaluate such strategies and effects on the publication rate at subsequent meetings.

## Conclusion

Free papers at the 2008 and 2010 ESSKA congress were published at a frequency that is comparable to that at other orthopaedic meetings. The publication rate was similar across all levels of evidence. Moreover, there was no association between the level of evidence of a paper and the impact factor of the journal in which it was published. Further encouragement of manuscript preparation and submission following these meetings could help to ensure important research findings are disseminated to large audiences.
